# Management of Locally Advanced or Metastatic Combined Hepatocellular Cholangiocarcinoma

**DOI:** 10.3390/cancers15030988

**Published:** 2023-02-03

**Authors:** Jemmy Zhao, Susann Stephan-Falkenau, Markus Schuler, Börge Arndt

**Affiliations:** 1 National Center of Tumor Diseases, German Cancer Research Center, Im Neuenheimer Feld 280, 69120 Heidelberg, Germany; 2 Institute of Pathology, Medizinisches Versorgungszentrum am Helios Klinikum Emil von Behring, Walterhöferstr. 11, 14165 Berlin, Germany; 3 Onkologischer Schwerpunkt am Oskar-Helene Heim, Clayallee 225a, 14195 Berlin, Germany; 4 Department of Hematology and Oncology, Helios Klinikum Emil von Behring, Walterhöferstr. 11, 14165 Berlin, Germany

**Keywords:** combined hepatocellular cholangiocarcinoma (cHCC-CC), neoadjuvant treatment, adjuvant treatment, locoregional treatment, palliative treatment, chemotherapy, sorafenib

## Abstract

**Simple Summary:**

Combined hepatocellular-cholangiocarcinoma is a rare and aggressive liver tumor that exhibits both hepatocytic and biliary differentiation. In this review we address the recent advances in the genetic and molecular characterization of this tumor and give an overview of possible therapeutic implications and systemic and locoregional treatment approaches of this tumor entity.

**Abstract:**

Combined hepatocellular cholangiocarcinoma (cHCC-CC) is a rare primary liver malignancy that comprises features of hepatocellular carcinoma (HCC) and cholangiocarcinoma (CC). Due to the rarity of this tumor, the treatment of choice has not yet been defined. For resectable disease, liver resection is the mainstay treatment. However, most patients relapse or display advanced disease and were not surgical candidates. Although the majority of patients are either primarily or secondarily treated in palliative intent, no guideline recommendations or prospective trial reports exist to allow reliable evaluation of debated treatment options. We review different locoregional or medical treatment options for advanced combined hepatocellular cholangiocarcinoma (cHCC-CC) in the neoadjuvant, adjuvant, or palliative setting and discuss the possibility of predictive biomarker-guided therapeutic options.

## 1. Introduction

Combined hepatocellular cholangiocarcinoma (cHCC-CC) is a rare primary liver malignancy displaying biphenotypic histomorphological and molecular characteristics of both hepatocellular carcinoma (HCC) and cholangiocarcinoma (CC) [1,2] In western countries, cHCC-CCs are reported to make up between 1% and 5% of all primary liver cancers [2], with an incidence of 0.05 per 100,000 persons per year [3]. Usually, the diagnosis of cHCC-CCs is based on routine histological investigations of small biopsy specimens or after surgical resection. The true prevalence of this tumor entity has likely been underestimated due to difficulties both in detecting histological subtypes in small samples and the lack of routine confirmation of HCC diagnosis by biopsy in patients with cirrhosis despite cirrhosis being present frequently (25–55%) in patients with cHCC-CC [3,4,5,6,7]. Epidemiological risk factors for cHCC-CC are similar to those of other primary liver cancers, (metabolic syndrome, excessive alcohol consumption, chronic hepatitis B and C infection, and liver cirrhosis), although significantly more patients with cHCC-CC had hepatitis B virus infection compared with CC, but the infection rate was similar to patients with HCC [8]. Liver surgery with lymph node dissection is the only curative option for patients with cHCC-CC and is considered the standard of care when feasible [9]. However, even after surgical interventions, tumor recurrence is frequent (up to 80% at 5 years), and 5-year survival rates do not exceed 30% [10,11,12]. Moreover, most patients with cHCC-CC are often diagnosed at an advanced stage, and only a minority of patients are suitable for surgical resection. Wakizaka et al. found that patients with cHCC-CC have higher blood levels of alpha-fetoprotein (AFP) and protein induced by vitamin K absence or antagonists-II than patients with CC. The prognosis of cHCC-CC and some pathological features, including vascular invasion and lymph node metastasis, were more similar to CC as compared with HCC. High carcinoembryonic antigen (CEA) levels and tumor size ≥ 5 cm were independent prognostic factors for both overall survival (OS) and recurrence in patients with cHCC-CC [8]. 

Compared with HCC and CC, the treatment of choice for advanced cHCC-CC has not yet been defined, and no standardized treatment protocols have been established to date. Clinical trials for either HCC or CC have excluded patients suffering from cHCC-CC. 

For advanced HCC, the multikinase inhibitor sorafenib was the standard of care for a decade, with a median OS of 10 to 12 months [13,14,15]. In 2018, lenvatinib was shown to be noninferior to first-line sorafenib in the phase III REFLECT trial. Median OS was 13.6 months in the lanvatinib group compared with 12.3 months in the sorafenib group. Furthermore, lenvatinib was better tolerated and achieved a higher objective response rate and delayed tumor progression [14]. In 2020, the IMbrave150 Trial showed that combined therapy with bevacizumab and atezolizumab improved OS as compared with front-line sorafenib monotherapy [16]. In the latest analysis of the IMbrave150 Trial, the median OS with the combined therapy was significantly better (19.2 versus 13.4 months), and the objective response rates were nearly threefold higher as compared with sorafenib (30 versus 11 percent) [17].

For locally advanced or metastatic CC, the combination of gemcitabine and cisplatin is the standard first-line chemotherapy, with median OS of 11.7 months [18]. In 2021, the TOPAZ-1 trial showed the superiority of adding the programmed cell death ligand 1 (PD-L1) antibody durvalumab to gemcitabine and cisplatin over gemcitabine/cisplatin alone. The median OS was improved significantly from 11.5 to 12.8 months in the Durvalumab group, and more than twice as many individuals were still alive at 24 months (24.9 versus 10.4 percent). The progression-free survival (PFS) and the overall response rate (ORR) were also improved with the addition of durvalumab [19]. In the absence of a standard treatment, clinicians often determine the dominant phenotype of tumor on the basis of radiologic characteristics and the presence of tumor markers, such as AFP or carbohydrate antigen 19-9 (CA 19-9), and recommend standard of care treatment for either HCC or CC. Furthermore, molecular characterization of the tumor should be strongly considered in patients with advanced stage cHCC-CC in order to identify potentially targetable genetic aberrations. 

### 1.1. Characterization of the Genomic Landscape of cHCC-CC and Possible Therapeutic Implications

Biomarker-assisted targeted therapy has recently made considerable progress in many entities in the field of oncology. Molecular analysis of tumor tissue may identify biomarkers that can aid in entity classification, have prognostic predictive value, and predictively guide therapeutic decisions. The characterization of the genomic landscape of cHCC-CC is closely related to the histological classification and the question of the origin of this bidirectionally differentiated entity. According to histological criteria established as early as 1949 by Allen and Lisa, the separate subtype with clearly demarcated tumor nodules showing purely hepatocellular or cholangiocellular differentiation can be distinguished from the combined subtype with intermingled tumor areas with both hepatocellular and cholangiocellular differentiation and the mixed subtype, the latter showing tumor cells with both differentiation features [1]. The 2010, the World Health Organization (WHO) classification included tumors with stem cell features in addition to the classic type of cHCC-CC, with the stem cell subtype showing cholangiocellular differentiation recently being reclassified as intrahepatic cholangiocarcinoma in the absence of a hepatocellular differentiated component [20]. The current WHO classification (2019) defines cHCC-CC as a primary liver carcinoma with the unequivocal presence of both hepatocytic and cholangiocytic differentiation within the same tumor on routine histopathology with hematoxilin eosin staining, regardless of the proportion of each component [21]. A “collision” tumor with physically separate and histologically different lesions, as previously subsumed under the separate subtype by Allen and Lisa, is, thus, excluded. Considering the difficulties in classifying this heterogeneous group of tumors histologically, it remains challenging to characterize the various tumor components genomically, especially in order to determine the cell of origin. It is a long-debated question and an active field of research whether primary liver cell tumors derive from mature hepatocytes or cholangiocytes or whether there is a malignant transformation of hepatic progenitor cells. It has been shown that mature hepatocytes and cholangiocytes develop from a bipotent stem/progenitor cell, termed hepatic progenitor cell or hepatoblast. In the regeneration process of severe liver injury, hepatoblasts with the ability to differentiate into hepatocytes and cholangiocytes can be detected in so-called stem cell niches within the portal field of the liver and are thought to originate from the terminal branches of the intrahepatic biliary system, the canals of Hering [22,23]. Moreover, adult hepatocytes in the repair process of chronic injury have the potential to dedifferentiate into stem-cell-like progenitors [24]. During this process, malignant transformation can occur, which is thought to be one mechanism for carcinogenesis of cHCC-CC [25]. Indeed, Wakizaka et al. showed that some cHCC-CCs express stem cell markers, such as Epithelial cell adhesion molecule (EPCAM), CD133, and CD56. The expression of these stem cell markers was more pronounced in the CC component than in the HCC component. Furthermore, expression of CD133 and EPCAM, but not CD56, in cHCC-CC was associated with poor prognosis. Overall survival and disease-free survival were significantly worse in patients with CD133- and EPCAM-positive tumors [26].

As reviewed by Aurélie Beaufrère et al. [27], several studies could demonstrate that hepatocyte progenitor cells are susceptible to malignant transformation into cHCC-CC in various mouse models [28,29,30]. However, mature hepatocytes with the ability to dedifferentiate have also been shown to undergo malignant transformation into cHCC-CC in cell culture and mouse models [24,31,32].

Coulouarn et al. performed a genome-wide transcriptional analysis of 20 histologically defined cHCC-CCs and reported that cHCC-CCs exhibit stem cell/progenitor features. Transforming growth factor beta and Wnt/β-catenin were identified as the two major signalling pathways activated in cHCC-CC. Interestingly, a β-catenin signature found in cHCC-CC was distinct from that observed in well-differentiated HCC with mutant β-catenin [33]. Moeini et al. found a significant correlation in the copy number variation of the CC and HCC components of the classical type of cHCC-CC, suggesting a clonal origin [34]. Sasaki et al., after DNA sequencing of 53 cHCC-CCs, postulated that there may be two pathways of histogenesis in cHCC-CC. One is that cHCC-CC arises as conventional HCC at an early stage and acquires biliary and/or stem cell features as it progresses. On the other hand, early-stage cHCC-CC arises as a biphenotypic carcinoma, suggesting hepatic progenitor cells as the cells of origin. As shown in Table 1, the most common mutations were TP53 (45.3%), TERT promoter mutation (31.3%), ARID1A (13.2%), IDH1/2 (11.8%), and KRAS (7.5%), defining four groups of tumors. First, the TERT-mutated group, which correlates with the intermediate subtype; second, the TP53-mutated-only subgroup; third, the group without mutation; and fourth, the ARID1A-mutated, KRAS-mutated, and IDH1/2-mutated groups [35], the latter correlating with the cholangiocellular subtype now classified as cholangiocarcinoma [21]. Using laser microdissection and whole-exome sequencing of 7 out of 15 cHCC-CCs, Wang et al. could show that cHCC-CCs have a large amount of ubiquitous nonsynonymous mutations and copy number variants (CNVs) common to HCC and CC, suggesting a monoclonal origin of cHCC-CC. As a challenge for targeted therapy, this study also demonstrated that cHCC-CCs contain substantial private mutations, ranging from 33.1 to 86.4%, as well as private somatic CNVs, indicating substantial intratumor heterogeneity. Seventy known driver mutation genes inclusive of TP53, MTOR, and ARID2 were identified, all of them linked to liver cancer carcinogenesis [36]. In order to define molecular targets for therapy, a molecular mapping of cHCC-CC seems to be urgently in need of further investigation. Considering tumor evolution, early common mutations should be addressed as molecular targets. In a recent large-scale study with 133 tumors using whole-exome and whole-genome sequencing, RNA sequencing, and single nucleus sequencing, Xue et al. demonstrated a monoclonal origin of the combined and mixed subtypes of cHCC-CCs (according to Allen and Lisa) and postulated these subtypes as distinct entities with different clinical and molecular characteristics [37]. The authors identified TP53, AXIN1, RB1, PTEN, ARID2, and BRD7 as significantly mutated genes, together with eight other recurrent genes reported in HCC and CC (TERT promoter, KMT2D, KEAP1, ARID1A, PTEN, RPS6KA3, CTNNB1, IDH1, PBRM1) as potential drivers in cHCC-CC (Table 1). The most frequently mutated driver genes were TP53 (49%) and the TERT promoter (23%, all hotspot C228T). In addition, other gene mutations were found that have been described rather rarely in primary hepatocellular tumors and are known to be associated with extracellular matrix formation and cell–cell adhesion (ADGRV1, MUC2, NEB, DST, and HMCN1), maintenance of nuclear and chromosomal integrity (SYNE1/2, SYCP2, and FRY), histone modification and DNA methylation (KMT2D, IDH1, BAP1, and EZH2), or chromatin remodeling (SWI/SNF, ARID1A, ARID2, PBRM1, and BRD7). In addition, KEAP1, IDH1, APOB, and ALB were mutated, suggesting that cellular energetics disruption may also be a feature of cHCC-CC. FGFR-related fusion events were identified in 6.5% of cases. Significant differences between histological subtypes were found only in AXIN1, which had more mutations in the mixed than in the combined subtype (2% in Com versus 20% in Mix; *p* < 0.001). Integrative analysis revealed that the combined type cHCC-CCs show strong CC-like features, such as higher expression of EPCAM, KRT19, and PRDM5, as well as enrichment of KRAS mutations and higher expression of KRAS, whereas the mixed type cHCC-CCs show HCC-like features, such as higher expression levels of AFP, GPC3, APOE, and SALL4, as well as a higher level of serum AFP. 

In a recent study, genomic profiling of cHCC-CC was used to train a machine learning (ML) model to classify a cHCC-CC case as CC-like or HCC-like in order to aid therapeutic decision-making. Of cHCC-CC cases, 16% (12/73) were ML-classified as CC-like, and 58% (42/73) cHCC-CC were ML-classified as HCC-like. The ML model classified more than 70% of cHCC-CC as CC-like or HCC-like on the basis of genomic profiles, without additional clinico-pathological input [38]. The most frequently altered genes in cHCC-CC were TP53 (65.8%), TERT (49.3%), and PTEN (9.6%) (Table 1). Within this cohort, 24.6% of tumors had genomic alterations that are linked to benefit from targeted therapies as BRCA2 (8.2%, 67% short variant, 25% were biallelic losses; 33% rearrangements), ERBB2 (5.5%, 75% amplifications), IDH1 (4.1%, 100% R132), BRAF (4.1%, 100% V600E), FGFR2 (4.1%, 67% fusions), and MET (2.7%, 100% amplifications) [38]. Yu Li Su et al. presented a case of a patient with advanced cHCC-CC, which harbored a clinically relevant single nucleotide variant of BRCA2, resulting in BRCA2 inactivation. The patient was treated with olaparib and achieved a remarkable regression of the tumor and a dramatically improvement of the laboratory values. The duration of response lasted for at least 7 months [39]. 

Although, no data are available regarding the benefit of ERBB2-, IDH1-, or FGFR2-directed therapies for patients with cHCC-CC, some data exist for their potential role in patients with biliary tract cancers. M. Javle et al. analyzed, in a retrospective cohort, the efficacy of ERBB2-directed therapy for 14 patients with ERBB2 amplification or overexpression suffering from gallblader cancer or cholangiocarcinoma. For all eight patients within the gallbladder cancer group, treatment was associated with disease control (one patient had a complete response (CR) and four patients a partial response (PR)). In contrast, no responses occurred in the cholangiocarcinoma group [40]. For patients who harbor IDH1-mutant cholangiocarcinoma and who had progressed on previous therapy, the Phase III ClarIDHy trial showed a meaningful clinical benefit for the IDH1-Inhibitor ivosidenib compared with placebo. PFS was significantly improved with ivosidenib compared with placebo and resulted in a favorable OS benefit, although, this was not statistically significant [41,42]. Abou-Alfa et al. and M. Javle et al. showed that the FGFR-Inhibitors Pemigatinib and Infigratinib had promising clinical activities in patients with locally advanced or metastatic cholangiocarcinoma harbouring FGFR2 gene fusions or rearrangements. With Pemigatinib, the ORR was 35.5%, and the median PFS was 6.9 months [43], while the objective response rate for infigratinib was 23.1%, and the median PFS was 7.3 months [44]. These study results reveal a high potential for precision therapy of cHCC-CC, an entity with comparatively poor prognosis and, so far, severely limited treatment options. Comprehensive molecular diagnosis of combined hepatocellular cholangiocarcinoma in routine diagnostics and presentation and discussion in an interdisciplinary molecular tumor board is recommended to assess prognostic and predictive biomarkers that guide therapeutic options.

### 1.2. Systemic and Locoregional Treatment Approaches for cHCC-CC: The Neoadjuvant Setting

As early symptoms for primary hepatic malignancies are lacking, and patients often develop locally advanced tumors, hence, putting up functional and anatomical challenges for curative surgical attempts, even in the absence of metastases. Despite this, little is known about the value of neoadjuvant treatment in combined hepatocellular cholangiocarcinoma.

There is currently only a single case report on primary irresectable cHCC-CC [45], where a large tumor with extensive locoregionary lymph node metastases showed remarkable metabolic and metric response after a neoadjuvant administration with six cycles of gemcitabine and cisplatin. The patient was subjected to surgery afterwards, and no adjuvant therapy was provided after resection margins were found to be tumor-free. The patient was reported to be disease-free after 15 months. Unfortunately, no further updates on the follow-up were published. 

Antwi et al. reported the outcome for patients with cHCC-CC after liver transplantation who underwent “neoadjuvant” locoregional therapy before transplantation [46]. It is important to note that before transplantation, all cases were classified as HCC and only the postresection pathological review displayed the biphenotypic histomorphological feature of cHCC-CC. Three different procedures were performed in the “neoadjuvant” setting for patients with cHCC-CC: transarterial chemoembolization (TACE), selective internal radiation therapy (SIRT), and radiofrequency ablation (RFA). Four patients showed complete response (21.1%), eight patients a partial response (42.1%), while only one patient displayed progressive disease. The disease control rate (DCR) of 94.7% was impressive for the locoregional therapy. The recurrence-free survival and the OS after liver transplantation were much better for patients who showed a partial or complete response compared with those who had stable disease or progressive disease. The 3-year OS was 92% for responders and only 43% for non-responders.

### 1.3. Systemic and Locoregional Treatment Approaches for cHCC-CC: The Adjuvant Setting

While surgical treatment is crucial to improving long-term prognosis of the aggressive malignancy, approximately half of the patients will experience early disease recurrence [47,48], thus reflecting a high demand for further treatment development. 

The literature on adjuvant therapeutic measurements is limited to a few case reports. This must be, in part, attributed to the relative rarity of cHCC-CC among primary liver tumors. Furthermore, the role of adjuvant therapy for either HCC or CC has remained elusive for decades. The STORM trial, a large double-blind placebo-controlled phase III trial, investigated sorafenib for HCC in an adjuvant setting and showed no benefit compared with the placebo arm. The BILCAP trial [49] is the first and, so far, only controlled randomized phase III trial to show a benefit in the adjuvant setting for biliary tract cancer despite not meeting its primary end point of improving overall survival. 

Uemura et al. [50] reported on a patient receiving adjuvant gemcitabine mono-therapy and yielding 20 months of disease-free survival before palliative treatment with S1 was administered (Table 2). 

Jou et al. recently published a report on another patient who was subjected to adjuvant gemcitabine and oxaliplatin (GEMOX) after liver segment resection and diagnosed to be tumor-free at 12 months follow-up [51]. 

Hayashi et al. [52] published a case report in 2006, presenting a multidisciplinary treatment of a young patient with a large primary lesion, including neoadjuvant TACE before surgical excision of the primary lesion. Afterwards, adjuvant therapy with cisplatin and 5-Fluorouracil (5-FU) was administered, and the patient was reported to be relapse-free for 42 months after surgery (Table 2). Miyata et al. performed a multidisciplinary approach, too, incorporating adjuvant radiation of irresectable hilar lymph node metastases and adjuvant hepatic arterial infusion and administered adjuvant Tegafur-Uracil for a decade. The patient was reported to be disease-free, even after an additional two years, totaling 12 years of disease-free survival so far [53]. Although there are no comparable experiences achieved, both cases highlight possible benefits of modality combination in an individualized matter to combat the general poor prognosis in curative treatment attempts if otherwise treated within adapted concepts of HCC or CC. Analogously to other solid tumors, risk factors have yet to be identified (e.g., degree of lymphatic metastasis, size of primary lesion, or quality of resection) to allow a risk-stratified treatment and its possible benefits, thereby surpassing the presently applied consensus-based decision-making.

### 1.4. Systemic and Locoregional Treatment Approaches for cHCC-CC: The Palliative Setting

There are only a few studies available that have investigated the role of TACE in the management of cHCC-CC. In a retrospective review of 50 cases with nonresectable disease, Kim et al. reported a DCR of 70% and a median OS of 12.3 months. Tumor response was significantly higher in patients with hypervascular tumors, while only one patient with a hypovascular tumor responded to TACE therapy [54]. In another retrospective study, Na et al. analyzed the efficacy of TACE in patients with relapse after surgical resection of the primary tumor. Interestingly, aligning with the findings of the previously sited study, the authors found that only patients with hypervascular tumors responded to TACE (ORR 36%) and had better OS rates than patients with hypovascular tumors [55]. Although only scant data are available that support the use of TACE therapy in patients with cHCC-CC, those with a hypervascular tumor seem to benefit, to some extent, from this locoregional therapy. In agreement with these data, the results of the retrospective study presented by Antwi et al. that was already discussed above, showed that patients with cHCC-CC who met radiological criteria for HCC with typical imaging features due to its hypervascularity had an impressive tumor response upon locoregional treatment [46]. Malone et al. evaluated the outcome of 22 patients who underwent SIRT with yttrium-90 and reported a disease control rate of 65%, with 3 patients harboring a CR (15%), 8 patients harboring a PR (40%), and 2 patients harboring a stable disease. After the treatment with radioembolisation, three patients were down-staged and suitable for surgical treatment. The median OS was 9.3 months in that patient cohort, suggesting a possible role for SIRT in cHCC-CC [56]. 

In contrast to HCC and CC, no guideline recommendations or prospective trial reports exist for the medical treatment of advanced cHCC-CC. Due to the rarity of this tumor, only retrospective observational studies are available with a limited number of patients [57,58,59,60,61]. At least five retrospective observational studies have analyzed the benefit of tyrosine kinase inhibitors (TKI) and platinum-based regimens for cHCC-CC. The results of these studies are summarized in Table 3. In a series of 55 evaluable patients who received systemic chemotherapy (gemcitabine and cisplatin/oxaliplatin n = 37; gemcitabine and 5-FU n = 13; or sorafenib n = 5) for advanced or metastatic cHCC-CC, 22% (11/55) in the chemotherapy group had an objective response, and 46% (23/55) achieved stable disease as the best response. In contrast, no patient in the sorafenib group achieved a partial response, and only one patient developed a stable disease. The highest DCR was achieved in the platinum-based therapy group (78.4%) compared with the gemcitabine and 5-FU group (38.5%) and the sorafenib group (20%). Median PFS was 8.0, 6.2, and 4.8 months for gemcitabine and platinum, gemcitabine and 5-FU, and sorafenib, and the median OS was 11.5, 11.7, and 9.6 months, respectively [59].

Another multicenter, retrospective study from Japan enrolled 36 patients who were treated with first-line platinum-based chemotherapy (gemcitabine and cisplatin (n = 12) and 5-FU and cisplatin (n = 11)), other chemotherapy regimens (n = 8), or with sorafenib (n = 5). At first evaluation, all five patients in the sorafenib group had progressive disease, and the median OS was only 3.5 months. In contrast, the median OS times for the platinum-based regimens were 11.9 (gemcitabine and cisplatin) and 10.2 months (5-FU and cisplatin). The authors concluded that the platinum-containing regimen had more favorable outcomes than the sorafenib treatment [58]. 

Gigante et al. conducted a retrospective analysis with a large cohort of 83 patients diagnosed with cHCC-CC who were treated with TKI or with systemic chemotherapy. Twenty-three patients were treated with sorafenib, one patient was treated with sunitinib, and another patient was treated with a combination of sunitinib and everolimus (TKI group). This group was compared with 54 patients treated with a platinum-based chemotherapy (5-FU and cisplatin n = 1; gemcitabine, oxaliplatin, and bevacizumab n = 5; gemcitabine and oxaliplatin n = 36; gemcitabine and cisplatin n = 11; and 5-FU and oxaliplatin n = 1). The data for response assessment were available from 68 patients out of 83 patients treated with first-line systemic therapy. Out of these 68 patients, 9 patients had a partial response (13%), and 36 patients had disease control (53%). The ORR was not significantly different between the TKI group (10%) and the platinum-based chemotherapy group (15.2%). In addition, the DCR was similar between these two groups (TKI group 45% vs. 58.7% for the chemotherapy group). In that cohort, the median OS was 11.9 months for the chemotherapy group and 8.3 months for the TKI-group, and the median PFS was 4.1 months for the chemotherapy group versus 2.8 months for the TKI group [61]. In this case, opposing the conclusion of the previous study, it was hypothesized that first-line systemic treatments with TKIs or platinum-based chemotherapies have similar efficacy in patients with unresectable/metastatic cHCC-CC. 

Another retrospective study from South Korea evaluated 99 patients who received systemic chemotherapy for histologically confirmed cHCC-CC unresectable or metastatic disease. From these 99 patients, 62 received sorafenib and 37 received chemotherapy. Among the 37 patients treated with systemic chemotherapy, most received platinum-containing regimens (n = 28), while fluoropyrimidine monotherapy and gemcitabine monotherapy were administered in 8 and 1 patients, respectively. Eight patients in the chemotherapy group achieved a PR (21.6%) as did six patients in the sorafenib group (9.7%). The DCR was similar between these two groups (48.8% and 54.8%) as was the median PFS (2.9 vs. 4.2 for the chemotherapy group vs. sorafenib group) and the median OS (10.6 months and 10.7 months). In a multivariate analysis, non-platinum-containing first-line chemotherapy was associated with poorer OS [60]. Thus, the results from this study indicate that sorafenib and chemotherapy have similar activity for this tumor entity. PFS and OS seem to be comparable to those expected of sorafenib for patients with HCC or chemotherapy for patients with CC. These data indicate, to some extent, the use of either treatment strategy (HCC-oriented vs. CC-oriented) depending on the dominant phenotype of tumor as first-line treatment for patients with combined HCC-CC. 

In a multicenter, retrospective study in France, oxaliplatin or the combination cisplatin and gemcitabine were prescribed for 30 patients (18 patients received gemcitabine and oxaliplatin, nine patients gemcitabine and cisplatin, and three patients GEMOX in combination with bevacizumab). Eight patients had a partial response (28.6%), another fourteen patients had stable disease, and six patients showed a progressive disease at first evaluation. The median OS was 16.2 months from time of diagnosis, and the PFS was 9.0 months [57].

In addition to these retrospective observational studies, several case reports have described a potential benefit of sorafenib, lenvatinib, checkpoint inhibitors, or different chemotherapy regimens.

A case from Japan reported on a patient with hepatitis-C-virus-induced liver cirrhosis and metastatic cHCC-CC who was treated with sorafenib. The patient achieved a CR on imaging after 6 months of treatment with sorafenib, and the duration of response lasted for at least 30 months [62]. 

Two case reports documented a possible activity of lenvatinib, which is a multikinase inhibitor of vascular endothelial growth factor receptor 1–3, fibroblast growth factor receptor 1–4, platelet-derived growth factor receptor α, stem cell factor receptor, and is approved as the first-line treatment for advanced HCC. Osuga et al. reported a case of a 77-year-old man who was treated with lenvatinib as a first-line therapy for unresectable combined hepatocellular-cholangiocarcinoma and described a meaningful response [63]. The dosage of lenvatinib in that case was 8 mg per day. The patient achieved partial response at 8 weeks follow-up, which has been maintained for at least 7 months after the initiation of lenvatinib. In the other case, lenvatinib was initiated as a third-line therapy, which yielded a mixed response after three months of treatment (partial response of brain metastasis but progressive bone metastasis) [64]. Another case report describes the efficacy of a PD-L1 inhibitor treatment in a patient presenting with metastatic cHCC-CC and overexpression of PD-L1. The patient showed an excellent clinical response and the duration of response lasted for at least 18 months [65]. N. Saito et al. presented another case report of a patient with unresectable cHCC-CC, which was treated successfully with atezolizumab and bevacizumab as third-line therapy. The patient received cisplatin and gemcitabine as first line- and lenvatinib as second line-therapy. Both therapies were stopped due to side effects and not due to progressive disease. With this therapy, the patient achieved a stable disease and a PFS of 7.5 months [66]. Rizell et al. currently report on administration of pembrolizumab that was given to a patient in the third-line therapy after limiting side effects under TKI and quick disease progression under cisplatin and gemcitabine occurred. The patient was successfully salvaged and displayed an encouraging complete remission after 6 months of pembrolizumab before treatment was stopped due to immune-related adverse events. The patient was reported to have no evidence of disease, even 24 months after treatment cessation. It is noteworthy that the tumor stained negative for PD-L1 and was microsatellite stable, thus questioning these markers as inclusion criteria for possible future clinical trials [67]. 

Hatano et al. reported on a patient that was treated with the oral fluoropyrimidine anticancer agent S-1 after tumor recurrence. The patient achieved a partial remission [68]. Chi et al. presented a 31-year-old Caucasian female who had a relapse of cHCC-CC within 10 months after surgical resection. The patient was successfully treated with gemcitabine in combination with cisplatin and achieved a partial remission, which lasted for 12 months [69]. 

The studies summarized above indicate that platinum-based regimens, such as gemcitabine and cisplatin or oxaliplatin regimens, are the most widely used and seem to be more promising than other chemotherapy regimens. However, their use is not supported by a high level of evidence. Whether TKI sorafenib or lenvatinib offer the same profit as platinum-based chemotherapy has to be evaluated in further clinical trials. The retrospective studies described above indicate at least some potential benefit of this therapy. In addition, the combination of checkpoint inhibitors, such as Atezolizumab or Durvalumab with platinum-containing regimens or even a quadruple regimen of PD-L1 inhibitors in combination with an anti-vascular endothelial growth factor therapy inhibitor and a platinum-based chemotherapy, could be a promising option. Such a quadruple combination has already been investigated for patients with metastatic nonsquamous NSCLC. The IMpower150 trial reported that the safety profile of this quadruple regimen was consistent with safety profiles of the individual drugs, and no new safety issues were identified with the combination [70]. The rationale for this idea is the combination of the standard treatment for either HCC or CC. However, to our knowledge, there are no clinical data available, even from case reports, supporting such a treatment regimen.

In the absence of a standard, evidence-based systemic treatment, genetic and molecular characterization of this tumor should be strongly considered to detect druggable genetic aberrations. For the future, clinical trials are warranted to optimize treatment strategies. However, there are multifactorial challenges to conducting clinical trials for this rare tumor type, such as late and incorrect diagnosis, logistical difficulties due to the very small patient populations, and lack of clinical expertise. However, these prospective trials are needed to identify the optimal management for unresectable cHCC-CC.

## 2. Conclusions

Combined hepatocellular-cholangiocarcinoma (cHCC-CC) is a rare and aggressive hepatic malignancy. Liver surgery with lymph node dissection is the only curative option for patients with cHCC-CC, however, tumor recurrence is frequent and the prognosis of cHCC-CC remains dismal. Whether neoadjuvant or adjuvant treatment regimens can improve prognosis has to be determined and no recommendation can be given for either treatments. 

For patients with advanced or metastatic disease there is no consensus regarding optimal systemic therapy. Platinum-based chemotherapies (especially in combination with gemcitabine) seem to be more promising than other chemotherapy regimens in respect to ORR, DCR and OS. Tyrosine kinase inhibition with sorafenib or lenvatinib are a therapeutic alternative, especially for patients with contraindications for chemotherapy. Locoregional treatment approaches, such as TACE or SIRT could be an additionally treatment option for patients with locally advanced and hypervascular tumor.

In the palliative setting, comprehensive molecular diagnosis should be performed to identify possible therapeutic targets for precision therapy of cHCC-CC.

## Figures and Tables

**Table 1:** Most common genes with alterations found in whole-genome/exome sequencing of combined hepatocellular cholangiocarcinoma.

Study, Reference:	Xue et al. [37]	Sasaki et al. [35]	Murugesan et al. [38]
Cohort size:	n = 133	n = 50	n = 73
TP53	49%	45.3%	65.8%
TERT promotor	23%	31.3%	49.3%
ARID1A	8%	13.2%	6.8%
RB1	8%	N/A	8.2%
IDH1	5%	11.8%	4.1%
CTNNB1	6%	N/A	6.8%
KRAS	N/A	7.5%	4.1%
AXIN1	10%	n/A	N/A
KMT2D	9%	N/A	N/A
KEAP1	8%	N/A	N/A
PTEN	7%	N/A	9.6%
HER2	N/A	N/A	4.1%
FGFR2	N/A	N/A	4.1%
BRAF	N/A	N/A	4.1%
MET	N/A	N/A	2.7%

TP53: Tumor protein p53; TERT: Telomerase-Reverse-Transcriptase; ARID1A: AT-rich interaction domain 1A; RB1: Retinoblastoma protein Transcriptional compressor 1; IDH: Isocitrate Dehydrogenase (NADP(+)) 1; CTNNB1: catenin beta-1; KRAS: Kirsten rat sarcoma virus; AXIN1: Axis inhibition protein 1; KMT2D: Lysin-Methyltransferase 2D; KEAP1: Kelch-like ECH-associated protein 1; PTEN: phosphatase and tensin homologue; HER2: human epidermal growth factor receptor 2; FGFR2: Fibroblast growth factor receptor 2; BRAF: B-Raf Proto-Oncogenen; MET: MET proto-oncogene; N/A: not available.

**Table 2:** Overview of published case reports on adjuvant systemic treatment for combined hepatocellular cholangiocarcinoma (cHCC-CC).

Study	Reference	Treatment	Result
Uemura et al., 2017	[50]	Gemcitabine	20 months disease-free survival
Jou et al., 2022	[51]	Gemcitabine and Oxaliplatin	12 months disease-free survival
Hayashi et al., 2006	[52]	Cisplatin and 5-Fluorouracil, neoadjuvant TACE	42 months disease-free survival
Miyata et al., 2019	[53]	Tegafur-Uracil, multidisciplinary HAI, and lymph node radiation	144 months disease-free survival

TACE: Transarterial chemoembolization; HAI: hepatic arterial perfusion.

**Table 3:** Overview of the largest published retrospective studies to date comparing treatment in advanced or metastatic hepatocellular cholangiocarcinoma.

Study	Reference	Treatment	Regimen/Agents	Cohort Size	Median OS	Median PFS
Gigante et al., 2022	[61]	TKI	Sorafenib	n = 23	8.3 mo	2.8 mo
		Chemotherapy	Platinum-based regimen	n = 54	11.9 mo	4.1 mo
Kobayashi et al., 2018	[58]	TKI	Sorafenib	n = 5	3.5 mo	N/A
		Chemotherapy	Cisplatin and Gemcitabine	n = 12	10.2 mo	3.0 mo
Kim et al., 2021	[60]	TKI	Sorafenib	n = 66	10.6 mo	2.9 mo
		Chemotherapy	n/A	n = 37	10.7 mo	4.2 mo
Salimon et al., 2018	[57]	Chemotherapy	GemOX, GemOX and Beva, Cisplatin and Gemcitabine	n = 30	16.2 mo	9.0 mo
Trikalinos et al., 2018	[59]	TKI	Sorafenib	n = 5	9.6 mo	4.8 mo
		Chemotherapy	Gemcitabine and Oxaliplatin/Cisplatin	n = 37	11.5 mo	8.0 mo
		Chemotherapy	Gemcitabine and 5-FU	n = 13	11.7 mo	6.2 mo

Median OS: Median overall survival; Median PFS: Median progression-free survival; TKI: Tyrosinekinaseinhibitor; GemOX: Gemcitabine and Oxaliplatin; Beva: Bevacizumab; 5-FU: 5-Fluorouracil; mo: month(s); N/A: not available.

## Abbreviations

5-FU	5-Fluorouracil
ADGRV1	adhesion g protein-coupled receptor V1
ALB	albumin
AFP	alpha-fetoprotein
APOB	apolipoprotein B
APOE	apolipoprotein E
ARID1A	AT-rich interaction domain 1A
ARID2	AT-rich interaction domain 2
AXIN1	axis inhibition protein 1
BRAF	B-Raf Proto-Oncogenen
BAP1	BRCA1-associated protein 1
BRD7	Bromodomain-containing 7
CA 19-9	carbohydrate antigen 19-9
CEA	carcinoembryonic antigen
CTNNB1	catenin beta-1
CC	cholangiocarcinoma
cHCC-CC	combined hepatocellular cholangiocarcinoma
CR	complete response
CNVs	copy number variants
DCR	disease control rate
DST	dystonin
EZH2	enhancer of zeste 2 polycomb repressive complex 2 subunit
EPCAM	epithelial cell adhesion molecule
FGFR2	fibroblast growth factor receptor 2
FRY	FRY microtubule binding protein
GPC3	glypican 3
HMCN1	hemicentin 1
HCC	hepatocellular carcinoma
HER2/ERBB2	human epidermal growth factor receptor 2/erb-b2 receptor tyrosine kinase 2
IDH	isocitrate Dehydrogenase (NADP(+))
KEAP1	kelch-like ECH-associated protein 1
KRT19	keratin 19
KRAS	kirsten rat sarcoma virus
KMT2D	lysin-Methyltransferase 2D
ML	machine learning
MTOR	mechanistic Target of Rapamycin
MET	MET proto oncogene
MUC2	mucin 2
NEB	nebulin
ORR	overall response rate
OS	overall survival
PR	partial response
PTEN	phosphatase and tensin homologue
PBRM1	polybromo 1
PD-L1	programmed cell death ligand 1
PFS	Progression-free survival
PRDM5	PR/SET domain 5
RFA	radiofrequency ablation
RB1	retinoblastoma protein Transcriptional compressor 1
RPS6KA3	ribosomal protein S6 kinase A3
SIRT	selective internal radiation therapy
SALL4	spalt-like transcription factor 4
SYNE1/2	spectrin repeat containing nuclear envelope protein ½
SWI/SNF	switch/sucrose non-fermentable
SYCP2	synaptonemal complex protein 2
TERT	telomerase-Reverse-Transcriptase
TACE	transarterial chemoembolization
TP53	tumor protein p53
TKI	tyrosine kinase inhibitors
WHO	World Health Organization
